# Orphan Nuclear Receptor Family 4A (NR4A) Members NR4A2 and NR4A3 Selectively Modulate Elements of the Monocyte Response to Buffered Hypercapnia

**DOI:** 10.3390/ijms25052852

**Published:** 2024-03-01

**Authors:** David E. Phelan, Ben Reddan, Masahiko Shigemura, Jacob I. Sznajder, Daniel Crean, Eoin P. Cummins

**Affiliations:** 1 School of Medicine, University College Dublin, Dublin 4, Irelandben.reddan@ucdconnect.ie (B.R.); 2 Conway Institute of Biomolecular and Biomedical Science, University College Dublin, Dublin 4, Ireland; 3 Division of Thoracic Surgery, Northwestern University, Chicago, IL 60611, USA; masahiko.shigemura@northwestern.edu; 4 Pulmonary and Critical Care Medicine, Feinberg School of Medicine, Northwestern University, Chicago, IL 60611, USA; j-sznajder@northwestern.edu; 5 School of Veterinary Medicine, University College Dublin, Dublin 4, Ireland

**Keywords:** hypercapnia, carbon dioxide, CO_2_, orphan nuclear receptor family 4A, NR4A2, NR4A3, RNA-seq, transcription

## Abstract

Hypercapnia occurs when the partial pressure of carbon dioxide (CO_2_) in the blood exceeds 45 mmHg. Hypercapnia is associated with several lung pathologies and is transcriptionally linked to suppression of immune and inflammatory signalling through poorly understood mechanisms. Here we propose Orphan Nuclear Receptor Family 4A (NR4A) family members NR4A2 and NR4A3 as potential transcriptional regulators of the cellular response to hypercapnia in monocytes. Using a THP-1 monocyte model, we investigated the sensitivity of NR4A family members to CO_2_ and the impact of depleting NR4A2 and NR4A3 on the monocyte response to buffered hypercapnia (10% CO_2_) using RNA-sequencing. We observed that NR4A2 and NR4A3 are CO_2_-sensitive transcription factors and that depletion of NR4A2 and NR4A3 led to reduced CO_2_-sensitivity of mitochondrial and heat shock protein (Hsp)-related genes, respectively. Several CO_2_-sensitive genes were, however, refractory to depletion of NR4A2 and NR4A3, indicating that NR4As regulate certain elements of the cellular response to buffered hypercapnia but that other transcription factors also contribute. Bioinformatic analysis of conserved CO_2_-sensitive genes implicated several novel putative CO_2_-sensitive transcription factors, of which the ETS Proto-Oncogene 1 Transcription Factor (ETS-1) was validated to show increased nuclear expression in buffered hypercapnia. These data give significant insights into the understanding of immune responses in patients experiencing hypercapnia.

## 1. Introduction

The levels of carbon dioxide (CO_2_) in the blood are closely regulated in the body through acute chemosensory mechanisms that respond to changes in the levels of CO_2_ and adjust the rate and depth of breathing. In certain lung diseases, e.g., chronic obstructive pulmonary disease (COPD), the partial pressure of CO_2_ in the blood can accumulate to levels >45 mmHg, termed hypercapnia. Hypercapnia is known to suppress immune and inflammatory signalling and is a risk factor in several diseases, e.g., COPD [1]. Hypercapnia can elicit signalling acutely through modulation of chemosensory and neuronal signalling and more chronically via changes in gene expression [2]. The mechanisms through which CO_2_ affects transcriptional responses are not fully understood [3] and may involve different transcription factors in different contexts. Work from our group and others indicates that multiple signalling pathways are modulated by increased CO_2_. However, relatively few transcription factors have been tested in interventional (e.g., knockdown/knockout) studies, which is the approach we have taken here to provide mechanistic insight.

The Orphan Nuclear Receptor Family 4A family members (NR4As) (*NR4A1-3*) are early response genes that are activated in response to a variety of stimuli and stressors [4,5] and modulate the differentiation of monocytes: classical to non-classical monocytes (*NR4A1*) and to dendritic cells (*NR4A3*). NR4A expression is associated with several pathologies, including arthritis [6,7], atherosclerosis [8], and cancer [9,10]. Regulation of NR4A activity is primarily at the transcriptional level, with a correlation between mRNA levels and activity. In recent years, several NR4A modulators have been developed and have demonstrated efficacy in numerous models, supporting the idea that these receptors are feasible therapeutic targets [11,12]. The NR4As can also undergo post-translational modifications, specifically phosphorylation and SUMOylation, which can also affect their activity [11,13,14]. We have recently characterised the transcriptional roles of *NR4A2* and *NR4A3* in THP-1 monocytes, revealing significant overlap of transcriptional targets as well as more selective antigen presentation (*NR4A2*-dependent) and viral response (*NR4A3*-dependent) roles [15].

The NR4A family of transcription factors and their role in the response to elevated CO_2_ are the focus of this study for a number of reasons. Firstly, nuclear hormone receptors, of which *NR4As* are members, represent a class of genes that was initially identified as being CO_2_ sensitive in *Caenorhabditis elegans* (*C. elegans*) [16]. In this seminal model organism study, E3 ligases, which regulate protein degradation via the ubiquitin pathway, and genes associated with the innate immune response were key classes of genes implicated in the C. elegans response to CO_2_ elevation. Subsequent to this study in worms, several studies have corroborated the role of the ubiquitin pathway [17,18] and immune regulation [19,20] in the CO_2_ response in mammalian systems. Indeed, a recent study identified ubiquitin as a carbon-dioxide-binding protein [21]. Thus, we hypothesised that nuclear receptor family members (which were robustly CO_2_ sensitive in *C. elegans*) are worth pursuing further as potential conserved CO_2_-sensitive transcriptional regulators. Furthermore, we and others have demonstrated a role for altered /suppressed Nuclear Factor of Kappa Light Chain Gene Enhancer in B cells (NFκB)-dependent signalling in the cellular response to hypercapnia [21,22,23]. The NR4As are known to be both regulated by NFκB-dependent signalling and capable of regulating NFκB-dependent transcription [24,25]. Thus, the NR4A family members have the potential to act at the intersection of NFκB-dependent and CO_2_-dependent signalling. In this study, we employ a hypothesis-directed approach to investigate the role of the NR4A family members *NR4A2* and *NR4A3* in the cellular transcriptional response to buffered hypercapnia in monocytes. Through the transcriptomic analyses in our short-hairpin ribonucleic acid (shRNA) system, we also infer core gene signatures and transcription factors that respond to hypercapnia stress, independently of *NR4A2/NR4A3* transcriptional effects.

## 2. Results

### 2.1. NR4A2 and NR4A3 Are CO_2_ Responsive in the Presence of LPS

**Figure 1:**
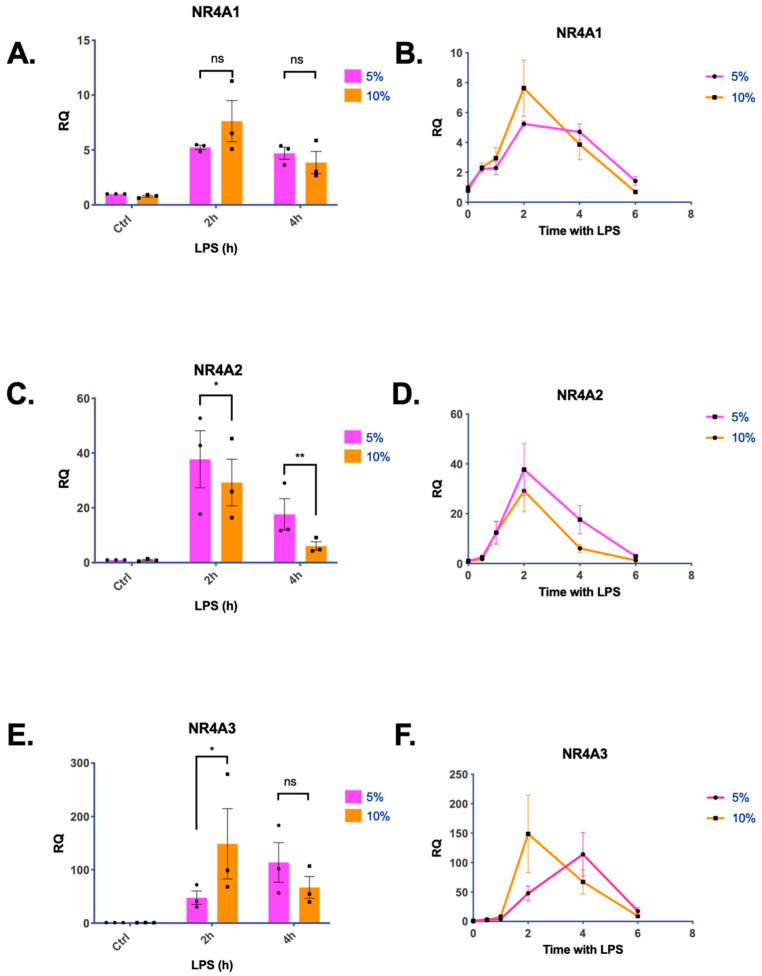
NR4A family members are sensitive to CO_2_ in THP-1 monocytes. Relative expression of *NR4A1* (**A**,**B**), *NR4A2* (**C**,**D**), and *NR4A3* (**E**,**F**) in THP-1 cells exposed to 5% or 10% CO_2_ for 8 h +/− LPS (2.5 μg/mL) for up to 6 h. *β-actin* was used as a housekeeping gene, and samples were normalised to a 5% CO_2_ untreated control. Data are presented as mean +/− Standard Error of the Mean (SEM) and are representative of *n* = 3 individual experiments. (**A**,**C**,**E**) show bar graphs including statistically significant differences using two-way Analysis of Variance (ANOVA), followed by Sidak’s multiple comparisons test (*p* < 0.05 denoted by *, *p* < 0.01 denoted by **. ns denotes no statistically significant difference, *p* > 0.05)). (**B**,**D**,**F**) display the same data visualised as a line graph over time.

To determine whether NR4A family members (1–3) are responsive to CO_2_, we exposed THP-1 monocytes to pre-equilibrated, buffered media in 5% or 10% CO_2_, for 8 h. For lipopolysaccharide (LPS)-treated cells, LPS was added 6, 4, 2, 1, or 0.5 h before the end of the experiment. Untreated cells were used as controls. All NR4A family members were temporarily induced by LPS. *NR4A1* had the lowest fold change increase in response to LPS, while *NR4A3* had the greatest inducibility. There were no statistically significant differences between 5% and 10% CO_2_ for *NR4A1* at these time points. *NR4A2* expression was significantly lower at 10% CO_2_ following 2 h and 4 h of LPS exposure. *NR4A3* expression was significantly higher in 10% CO_2_ at 2 h of LPS exposure compared to 5% CO_2_ (Figure 1A–F). Taken together, these data indicate that elevated CO_2_ exposure shifts the temporal response of *NR4A2* and *NR4A3* to LPS stimulation. This effect is most evident at 2 h and 4 h of LPS treatment (Figure 1C,E). The CO_2_-dependent impact on LPS-induced NR4A expression was also evident, with decreased protein levels of NR4A2 in THP-1 cells exposed to buffered media in 5% or 10% CO_2_ +/− LPS (Supplementary Figure S1).

### 2.2. The Cellular Response to Elevated CO_2_ Is Modulated by shNR4A2

Given the evidence that elevated CO_2_ modulates the transcriptional kinetics of *NR4A2* and *NR4A3* against a background of inflammation, we hypothesised that NR4A2 and NR4A3 may play roles as master regulators of the monocyte response to hypercapnia. To test this, we took an interventional approach using cells that are stably depleted of *NR4A2* and *NR4A3* using targeted shRNAs. The consequences of shNR4A2 and shNR4A3 in normocapnic monocytes [15] have recently been published. Here we report for the first time on the transcriptional response to elevated CO_2_ in (short hairpin RNA targeting *NR4A2*) shNR4A2 and (short hairpin RNA targeting *NR4A3*) shNR4A3 cells, with comparisons to some of our previously published work. This approach is well suited to test our hypothesis that NR4A family members are regulated by hypercapnia and contribute to the cellular response to hypercapnia.

**Figure 2:**
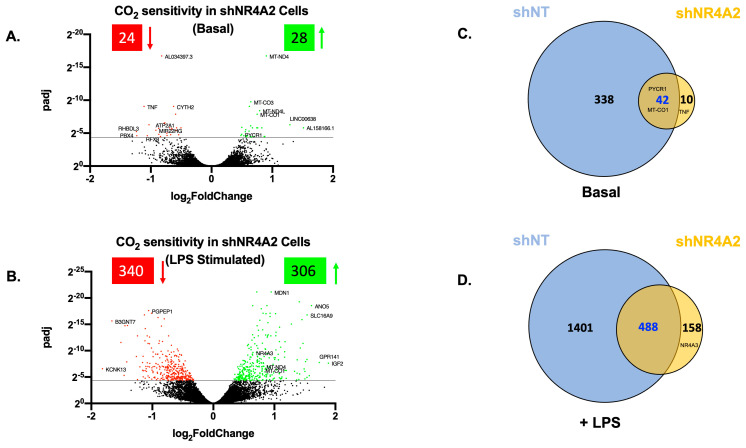
Hypercapnia regulates gene expression in *NR4A2*-depleted monocytes in the presence and absence of LPS. Volcano plot of differential expression between 5% and 10% CO_2_ (4 h) in shNR4A2 THP-1 cells in the absence (**A**) and presence (**B**) of LPS (2.5 μg/mL for 2 h). The cut-off for significance (adjusted *p*-value (*p*-adj) < 0.05) is shown with a solid line on the *y*-axis. Significantly upregulated genes are shown in green, and downregulated genes are shown in red. Venn diagram representing the number of genes differentially expressed in hypercapnia (4 h) in shNT (blue) and shNR4A2 (orange) THP-1 cells in the absence (**C**) and presence (**D**) of LPS (2.5 μg/mL for 2 h). Data is representative of *n* = 3 individual experiments with examples included for each group.

We first investigated the CO_2_-responsiveness of shNR4A2 cells, both in the basal and LPS-stimulated states. Buffered hypercapnia (10% CO_2_) for 4 h was a modest stimulus in these cells, with 52 transcripts differentially expressed in the basal state (Figure 2A, Supplementary Table S1) and 646 transcripts in the LPS-stimulated cells (Figure 2B, Supplementary Table S2). Interestingly, there were markedly fewer transcripts in shNR4A2 cells compared to non-target short hairpin RNA (sh)-controls ((380 genes (basal) and 1889 genes (LPS-stimulated)) [26], suggesting that the depletion of *NR4A2* attenuated the cellular response to hypercapnia. Of the 52 genes affected by hypercapnia in the basal state, 42 (or 80%) were also affected by hypercapnia in sh-control cells (Figure 2C). Of the 646 genes affected by hypercapnia in the presence of LPS, 488 (or 75%) were also affected by hypercapnia in non-target control cells (Figure 2D). Taken together with the principal component analysis Supplementary Figure S2A,B, indicate that (i) buffered hypercapnia is a robust stimulus to change gene expression in shNR4A2 cells, (ii) that the cellular response to hypercapnia is enhanced in the presence of LPS, and (iii) that the total number of transcripts affected is fewer than those seen in non-target control cells in the presence or absence of LPS. The top ten transcripts most affected by 10% CO_2_ (up and downregulated, ranked by fold change) are included in Supplementary Figure S3.

### 2.3. The Cellular Response to Elevated CO_2_ Is Modulated by shNR4A3

**Figure 3:**
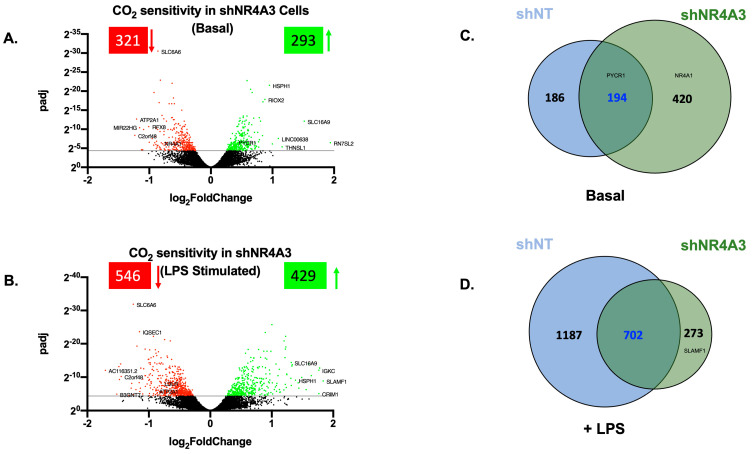
Hypercapnia regulates gene expression in NR4A3-depleted monocytes in the presence and absence of LPS. Volcano plot of differential expression between 5% and 10% CO_2_ (4 h) in shNR4A3 THP-1 cells in the absence (**A**) and presence (**B**) of LPS (2.5 μg/mL for 2 h). The cut-off for significance (*p*-adj < 0.05) is shown with a solid line on the *y*-axis. Significantly upregulated genes are shown in green, and downregulated genes are shown in red. Venn diagram representing the number of genes differentially expressed in hypercapnia (4 h) in shNT (blue) and shNR4A3 (green) THP-1 cells in the absence (**C**) and presence (**D**) of LPS (2.5 μg/mL for 2 h). Data is representative of *n* = 3 individual experiments with examples included for each group.

We next investigated the CO_2_-responsiveness of shNR4A3 cells, both in the basal and LPS-stimulated states. Buffered hypercapnia (10% CO_2_) for 4 h was a robust stimulus in these cells, with 614 transcripts differentially expressed in the basal state (Figure 3A, Supplementary Table S3) and 975 transcripts in the LPS-stimulated cells (Figure 3B, Supplementary Table S4). Interestingly, this was markedly more transcripts than in sh-control and shNR4A2 cells in the basal state, suggesting that the depletion of *NR4A3* modulated the cellular response to hypercapnia somewhat differently than the depletion of *NR4A2*. Of the 614 genes affected by hypercapnia in the basal state, 194 (or 31%) were also affected by hypercapnia in non-target control cells (Figure 3C). Of the 975 genes affected by hypercapnia in the presence of LPS, 702 (or 72%) were also affected by hypercapnia in non-target control cells (Figure 3D). Taken together with the principal component analysis, Supplementary Figure S2C,D, indicate (i) that buffered hypercapnia is also a robust stimulus to change gene expression in shNR4A3 cells; (ii) that the cellular response to hypercapnia is enhanced in the presence of LPS; and (iii) that the total number of transcripts affected is greater than those seen in non-target control cells in the basal state and fewer in the presence of LPS under the same conditions. The top ten transcripts most affected by 10% CO_2_ (up and downregulated, ranked by fold change) are included in Supplementary Figure S4.

### 2.4. Comparison of CO_2_ Responsiveness in Cells Depleted of NR4A2 and NR4A3

**Figure 4:**
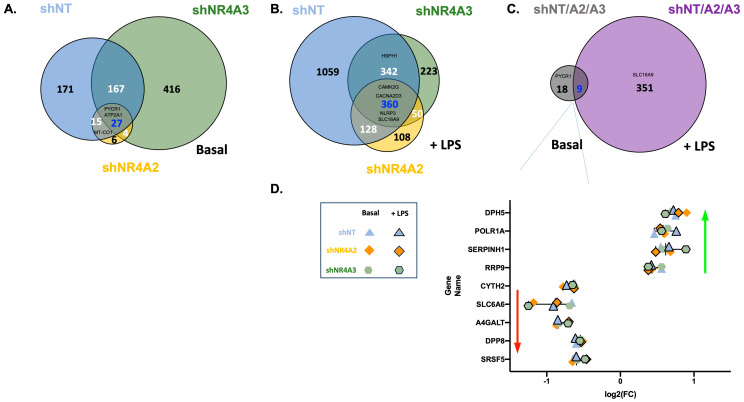
Comparison of CO_2_-sensitive genes in shNT, shNR4A2, and shNR4A3 THP-1 monocytes. Venn diagram representing the number of genes differentially expressed in hypercapnia (4 h) in shNT (blue), shNR4A2 (orange), and shNR4A3 (green) THP-1 cells in the basal state. Representative examples are included for each group (**A**). Venn diagram representing the number of genes differentially expressed in hypercapnia (4 h) in shNT (blue), shNR4A2 (orange), and shNR4A3 (green) THP-1 cells in the presence of LPS (2.5 μg/mL for 2 h). Representative examples are included for each group (**B**). Venn diagram representing the number of genes differentially expressed in hypercapnia (4 h) in shNT/shNR4A2/shNR4A3 THP-1 cells in the basal (grey) and LPS stimulated (purple) (2.5 μg/mL for 2 h) states. Representative examples are included for each group (**C**). A forest plot represents the differential expression of 9 genes that were CO_2_ responsive regardless of NR4A expression and LPS stimulation (**D**). Data is representative of *n* = 3 individual experiments.

The data from Figure 2 indicates a significant overlap in transcripts affected by elevated CO_2_ in both non-target cells and shNR4A2 cells. Commonly regulated genes include those associated with mitochondrial gene expression, e.g., Mitochondrially Encoded Cytochrome C Oxidase 1 (*MT-CO1*), Mitochondrially Encoded NADH: Ubiquinone Oxidoreductase Core Subunit 4 (*MTND-4*), Mitochondrially Encoded NADH: Ubiquinone Oxidoreductase Core Subunit 4L (*MT-ND4L*) (Supplementary Figure S6) and genes involved in cellular metabolism Pyrroline-5-Carboxylate Reductase 1 (*PYCR1*) (Figure 4A,C). Genes associated with immune signalling, e.g., Tumour Necrosis Factor (*TNF*) (Figure 2C and Supplementary Figure S7B) and *NR4A3* (Figure 2D and Supplementary Figure S9C) were selectively CO_2_ sensitive in shNR4A2 cells. Similarly, the data from Figure 3 indicates a significant overlap of transcripts affected by CO_2_ in both non-target cells and shNR4A3 cells. Commonly regulated genes include those associated with cellular metabolism (*PYCR1*) (Figure 4A,C); however, the signature for mitochondrial and metabolism-associated genes is less striking in shNR4A3 cells (Supplementary Figure S8). Genes associated with immune signalling, e.g., *NR4A1* (Figure 3C, Supplementary Figure S9A) and Signalling Lymphocytic Activation Molecule Family Member 1 (*SLAMF1*) (Figure 3D, Supplementary Figure S4B) were selectively CO_2_ sensitive in shNR4A3 cells.

From our comparisons of the CO_2_ sensitivity of shNR4A2 cells and shNR4A3 cells compared to shNT cells, we observed several genes whose response to hypercapnia was conserved in *NR4A2* or *NR4A3*-deficient cells, as well as genes that were uniquely sensitive to CO_2_. Examination of RNA-sequencing (RNA-seq) transcript per million (TPM) data from the uniquely sensitive transcripts revealed that while some transcripts failed to reach the threshold for CO_2_ sensitivity, the mean trends were qualitatively similar to those observed in the other cell types, e.g., *TNF* is selectively decreased in response to CO_2_ in the basal state in shNR4A2 cells; however, a decrease is also observed in shNT cells and shNR4A3 cells (albeit non-significantly) (Supplementary Figure S7B). Similarly, there were transcripts that were clearly affected by shNR4A2 on a basal level, e.g., Wnt Family Member 7B (*WNT7B*), but retained an equivalent level of CO_2_ sensitivity (Supplementary Figure S7A). Thus, the data generated to date indicates that NR4A family members *NR4A1-3* are CO_2_-sensitive transcripts in monocytes and selectively modulate elements of the monocyte response to buffered hypercapnia without being master regulators.

#### Many Genes Are Commonly Regulated by CO_2_ in shNT, shNR4A2, and shNR4A3 Cells

We next exploited the similarities in the responses of our three different cell lines to elevated CO_2_. We reasoned that transcripts that were consistently CO_2_ sensitive across three genetically modified THP-1 cell lines (with very different basal transcription of 100s of genes due to shNR4A2 or shNR4A3 [15]) represent particularly robustly CO_2_ responsive transcripts. These data relating to core CO_2_-responsive genes can enhance our understanding of the cellular response to hypercapnia in monocytes with and without LPS treatment.

In the basal state, 27 genes were commonly differentially expressed across all 3 groups (Figure 4A). These included genes involved in cellular metabolism (*PYCR1*, Aldehyde dehydrogenase 1 Family Member B1 (*ALDH1B1*)), regulation of calcium (Cluster of Differentiation 38 (*CD38*), Sarcoplasmic/Endoplasmic Reticulum Calcium ATPase1 (*ATP2A1*), and serpin peptidase inhibitors Serpin Family B Member 2 (*SERPINB2*) and Serpin Family H Member 1 (*SERPINH1*)). The mitochondrial oxidative phosphorylation (OXPHOS) genes (*MT-CO1, MT-CO2, MT-CO3, MT-ND4, MT-ND4L*, and *MT-ND5*) were significantly differentially expressed in hypercapnia in shNT and shNR4A2 cells, but not in shNR4A3 cells in the basal state.

In the presence of LPS, there were 360 common differentially expressed genes (DEGs) across all 3 groups (Figure 4B). This included genes involved in inflammation (Nucleotide-Binding Oligomerisation Domain, Leucine Rich Repeat and Pyrin Domain Containing 3 (*NLRP3*)), calcium signalling (Calcium/Calmodulin Dependent Protein Kinase II Gamma (*CAMK2G*), Calcium Voltage-Gated Channel Auxiliary Subunit Alpha-2/Delta-3 (*CACNA2D3*)) and solute carriers (e.g., Solute Carrier Family 6 (Neurotransmitter Transporter, Taurine), Member 6 (*SLC6A6*), Solute Carrier Family 16 (Monocarboxylic Acid Transporters), Member 9 (*SLC16A9*), Solute Carrier Family 17 (Acidic Sugar Transporter), Member 5 (*SLC17A5*), Solute Carrier Family 39 (Metal Ion Transporter), Member 8 (*SLC39A8*), Solute Carrier Family 39 (Metal Ion Transporter), Member 14 (*SLC39A14*)). While some of the chaperone proteins were common to all three groups (e.g., Heat Shock Protein 90 Alpha Family Class A Member 1 (*HSP90AA1*), Heat Shock Protein Family A (*HSP70*) Member 1A (*HSPA1A*) (Supplementary Figure S7C), DNAJ Heat Shock Protein Family (*Hsp40*) Member A1 (*DNAJA1*)), others were sensitive in shNT and shNR4A3 cells, but not shNR4A2 (e.g., Heat Shock Protein Family H (Hsp110) Member 1 (*HSPH1*) (Supplementary Figure S7D), Heat Shock Protein Family A(HSP70) Member 4 (*HSPA4*)).

Finally, we compared the 27 genes commonly differentially expressed in hypercapnia in the basal state with the 360 common DEGs in the presence of LPS. The resulting list had nine common genes. These genes followed strikingly similar patterns of differential expression in response to hypercapnia (Serine and Arginine Rich Splicing Factor 5 (*SRSF5*), Dipeptidyl Peptidase 8 (*DPP8*), Alpha 1,4,-Galactosyltransferase (*A4GALT*), *SLC6A6*, Cytohesin 2 (*CYTH2*) downregulated (Figure 4D, Supplementary Figure S10A–E), (Ribosomal RNA Processing 9, U3 Small Nucleolar RNA Binding Protein (*RRP9*), Serpin Family H Member 1 (*SERPINH1*), RNA Polymerase 1 Subunit A (*POLR1A*), Dipthamide Biosynthesis (*DPH5*) upregulated (Figure 4D, Supplementary Figure S10F–I), regardless of LPS or *NR4A2/NR4A3* depletion, thus representing a cluster of fundamental gene signatures in response to elevated CO_2_.

### 2.5. ETS-1 Is a CO_2_ Responsive Transcription Factor in Monocytes

To better understand the transcriptional regulation of transcripts in response to elevated CO_2_ in monocytes, we performed MetaCore Transcriptional Analysis to determine the most interacted transcription factors with CO_2_-dependent transcripts in shNT-, shNR4A2, and shNR4A3 cells + LPS. The most interacted transcription factors are ranked by z-score in Supplementary Table S5. The most consistently CO_2_ dependent interacted transcription factors with the highest z-scores were RUNX Family Transcription Factor 1 (AML1), SRY (Sex Determining Region Y)-Box 17 (SOX17), T-Cell Acute Lymphocytic Leukaemia Protein 1 (TAL1), ETS-1, and LIM Domain Only 2 (LMO2).

**Figure 5:**
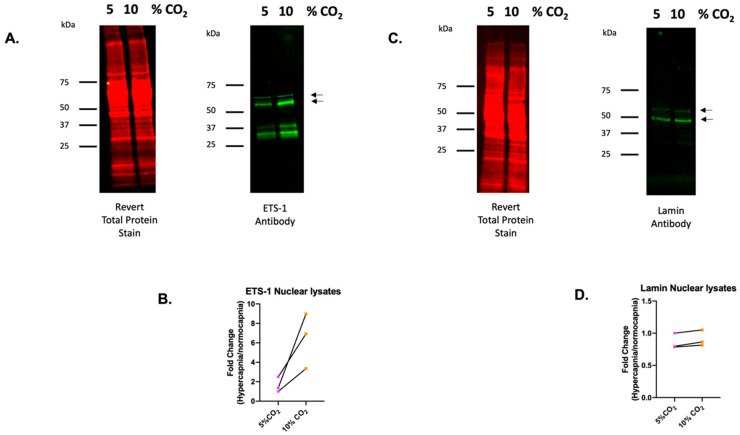
ETS-1 nuclear expression is increased in hypercapnia. Western blot analysis of nuclear lysates from THP-1 monocytes exposed to 5% or 10% CO_2_ for 24 h. Lysates were probed using a revert total protein stain and imaged in the 700 nm (red) channel or incubated with an (**A**) ETS-1 or (**C**) Lamin primary antibody followed by a fluorescent secondary mouse antibody and imaged in the 700 nm (red) or 800 nm (green) channel on an Li-COR imaging system. The image is representative of 3 independent experiments. Protein quantification was performed using EmpiriaStudio and plotted as fold change (Hypercapnia/normocapnia) for protein expression of (**B**) ETS-1 and (**D**) Lamin normalised to total revert stain intensity. Arrows indicate the bands on the western blot that were quantified for protein expression.

To narrow our search for associated transcription factors further, we next interrogated the EnCyclopedia of DNA Elements (ENCODE) project website (www.encodeproject.org) with nine fundamental gene signatures in response to CO_2_ (Figure 4D, Supplementary Figure S10), and investigated the evidence for regulation of these genes by our five highest scoring transcription factors. Interestingly, we identified ETS-1 as a putative transcriptional regulator of all nine CO_2_-sensitive genes in Figure 4D. For this reason, we next focused our attention on ETS-1. Given that ETS-1 is a nuclear transcription factor, we investigated the CO_2_-sensitivity of this protein in monocytes exposed to normocapnia or hypercapnia under pH-buffered conditions. Western blot analysis of nuclear lysates revealed a CO_2_-dependent increase in the nuclear expression of the ETS-1 protein, supporting the concept that this transcription factor is involved in CO_2_-dependent gene expression (Figure 5A,B). Lamin (acting as a control for nuclear protein expression) was not changed by the exposure to CO_2_ under the same conditions (Figure 5C,D). Taken together, these data further implicate ETS-1 as a novel CO_2_-responsive transcription factor in monocytes.

## 3. Discussion

The primary aim of this study was to investigate the role of NR4A2 and NR4A3 in CO_2_-dependent signalling in monocytes. As discussed in the introduction, we hypothesised that these members of the nuclear receptor family may represent master regulators of the cellular response to CO_2_. Our initial experiments demonstrated a temporal response of NR4A family members to LPS (Figure 1). All three NR4A family members were rapidly induced at the transcript level in response to LPS, with *NR4A3* showing the highest level of induction relative to controls. The rapid induction of NR4A mRNA has previously been demonstrated in THP-1 monocytes, with similar observations regarding *NR4A3* [27,28]. We noted various sensitivities of the different NR4A family members to CO_2_ concentrations in THP-1 cells. *NR4A1* showed the least sensitivity. *NR4A2* expression was reduced in hypercapnia, with lower expression at both 2 h and 4 h of LPS stimulation in 10% CO_2_ compared to 5% CO_2_. For *NR4A3*, the maximal increase in NR4A3 mRNA expression in response to LPS shifted from 4 h at 5% CO_2_ to 2 h at 10% CO_2_. Taken together, these data suggest a CO_2_-dependent shift in the temporal response of NR4A family members *NR4A2* and *NR4A3* to LPS (Figure 1). This is associated with a reduction in NR4A2 protein levels in hypercapnia (Supplementary Figure S1). Interestingly, microarray data from human macrophages stimulated with LPS found *NR4A2* to be the most highly upregulated gene following a 90-minute exposure to hypercapnia-acidosis (20% CO_2_) [20]. While *NR4A2* was not the focus of the Casalino-Matsuda study, it further supports the idea that environmental CO_2_ conditions shape the cellular response to inflammatory stimuli.

Given the CO_2_ sensitivity of *NR4A2* and *NR4A3* to CO_2_ in this model, we investigated the impact of depletion of these genes in our model of buffered hypercapnia. In the basal state, depletion of *NR4A2* decreased the number of genes significantly differentially expressed in hypercapnia compared to shNT cells (Figure 2A,C), while depletion of *NR4A3* increased this number in the basal state (Figure 3A,C). This could suggest that in the basal state, *NR4A2* has a role in the regulation of CO_2_-sensitive genes, while *NR4A3* may act to attenuate the effect of hypercapnia on gene expression. The difference in CO_2_ sensitivity seen in shNR4A2 and shNR4A3 cells in the absence of LPS is somewhat surprising, as the NR4As are expressed at low levels in the basal state. In the presence of LPS, both shNR4A2 and shNR4A3 cells had fewer significant DEGs in response to hypercapnia compared to shNT cells (Figure 2B,D and Figure 3B,D). It is possible that the different pattern of CO_2_ sensitivity observed between the basal and LPS states may be due to the nature of the knockdown. While *NR4A2* and *NR4A3* mRNA expression were clearly depleted in shNR4A2 and shNR4A3 cells, respectively, they were still capable of being increased by LPS exposure (Supplementary Figure S9). Taken together, these data indicate that the level of *NR4A2* and *NR4A3* in monocytes affects the cellular response to CO_2_. In the case of shNR4A2 cells in the basal state, it is interesting to note that *TNF*, a known CO_2_-sensitive transcript in lung cells [29], is only significantly affected in shNR4A2 cells. NR4A2 is known to act as a suppressor of NFκB and inflammatory signalling [25]. The change in the sensitivity of *TNF* under these particular conditions may reflect a more pro-inflammatory milieu in shNR4A2 cells. Thus, the cellular microenvironment, which can be influenced by inflammatory signalling and metabolic changes, is likely a key determinant in shaping the cellular response to CO_2_. This concept is further supported by our RNA-seq data indicating that (increased) *NR4A3* is only sensitive to CO_2_ in shNR4A2 cells in response to LPS (Figure 2D, Supplementary Figure S9C) and that (decreased) *NR4A1* is only sensitive to CO_2_ in shNR4A3 cells in the basal state (Figure 3C, Supplementary Figure S9A). Therefore, the summation of CO_2_ signalling, and the cellular microenvironment of inflammation/ metabolism ultimately dictate the transcriptional response.

We have previously published a strong signature for increased mitochondrial transcription in response to acute hypercapnia in monocytes [26]. When comparing the CO_2_ sensitivity of shNR4A2 cells to shNT cells, this mitochondrial signature was preserved, although there were much fewer DEGs identified. This mitochondrial signature is reflected both in the significant differential expression of mitochondrial OXPHOS genes, as well as in the GO terms associated with hypercapnia in shNR4A2 cells (Supplementary Figure S8). Interestingly, when analysing the CO_2_ sensitivity of shNR4A3 cells, the mitochondrial signature seems to be much reduced (Supplementary Figure S8). While there were more significantly differentially expressed genes in hypercapnia in shNR4A3 cells in the basal state, none of the mitochondrial genes reached the threshold for significance (Supplementary Figure S6). Furthermore, Mitochondrial Transcription Factor 1 (TFAM) interacted with shNT and shNR4A2 cells but not shNR4A3 cells in hypercapnia (+LPS) (Supplementary Table S5), further supporting the concept that the mitochondrial response to CO_2_ is blunted in shNR4A3 cells. The NR4As have been shown to regulate genes associated with fuel utilisation [5,30,31]. In addition, *NR4A3* knockdown in C2C12 myotubes reduces mitochondrial health and mRNA levels of key mitochondrial genes, e.g., Peroxisome Proliferator-Activated Receptor Gamma Coactivator 1-Alpha (*PGC1α*) and *TFAM*, as well as the protein levels of mitochondrial complexes I–IV [32]. Thus, a specific role for NR4A3 in the regulation of mitochondrial and metabolic pathway genes may account for the lack of sensitivity of these pathways to hypercapnia in shNR4A3 monocytes.

shNR4A2 cells appear to differ in their response to hypercapnia with respect to heat shock proteins (Hsps). Hsps are induced in response to cellular stress and protect the cell through various functions, including maintaining proteostasis by chaperoning misfolded proteins and assisting with antigen presentation [33,34]. Hsp induction is highly conserved and is regulated by the transcription factor heat shock factor 1 (HSF1). Hypercapnia has previously been reported to alter the activity of HSF1 in immune cells, with increased HSF1 protein expression and nuclear localisation observed in murine alveolar macrophages after exposure to 15% CO_2_ for 16 h [35]. Furthermore, mass spectrometry has identified Hsp70 as a major NR4A-interacting protein in human embryonic kidney cells (HEK293) [36]. The loss of Hsp sensitivity to CO_2_ in NR4A2-depleted cells (Supplementary Figure S7D), as well as the loss of CO_2_-responsive GO terms associated with protein folding and ribosome biogenesis (Supplementary Figure S8), indicates that *NR4A2* is responsible, at least in part, for the regulation of certain genes in hypercapnia.

Taken together, our data provide evidence of selective regulation of CO_2_-dependent transcripts in shNR4A2 and shNR4A3 cells with regard to mitochondrial genes and Hsp-related genes, respectively. In addition, our data provides evidence of many CO_2_-sensitive genes being relatively insensitive to depletion of *NR4A2* and *NR4A3*, indicating that other conserved transcription factors are involved in the transcriptional response to buffered hypercapnia in this model. There is a known redundancy among NR4A family members [10], meaning it is possible that knockdown of one isoform can be compensated for by the remaining other isoforms.

To gain insight into other potential CO_2_-sensitive transcription factors involved in the transcriptional response to buffered hypercapnia, we next focused on transcriptional signatures that were common to shNT, shNR4A2, and shNR4A3 cells. We reasoned that transcripts that were consistently CO_2_-sensitive across the three different cell lines represent particularly robustly CO_2_ sensitive transcripts. Data in Figure 4 reveals a striking overlap of CO_2_-sensitive genes in cells that have quite different basal transcriptional profiles [15]. Indeed, nine genes were CO_2_-sensitive in all three cell lines, both with and without LPS stimulation. The consistency of the response is evident in Figure 4D and Supplementary Figure S10, with four transcripts consistently upregulated in hypercapnia (*RRP9, DHP5*, *SERPINH1*, and *POLR1A*) and five transcripts consistently downregulated (*SRSF5, CYTH2, A4GALT, DPP8*, and *SLC6A6*). These genes are associated with RNA metabolism (*SRSF5, POLR1A*), metabolite conversion (*A4GALT*), protein modification (*DPP8*), and protein-binding activity (*SERPINH1*, *CYTH2*), with no obvious initial common link apparent. Taken together, these data speak to the responsiveness of multiple important, diverse pathways to buffered hypercapnia.

In order to gain further insight into CO_2_-sensitive transcriptional regulators, we sought to exploit our knowledge regarding genes commonly regulated by CO_2_ across our three cell lines. Metacore transcription factor analysis revealed a notable conservation of interactions between several transcription factors, including AML1, SOX17, TAL1, ETS1, and LMO2 (Supplementary Table S5). Transcription factors previously linked with hypercapnia were also identified in our list but were not among the most prominent hits in our model, e.g., Transcription Factor P65 (RelA) [29], NFκB Subunit 2 (NFKB2) [22], Hypoxia Inducible Factor 1 Alpha (HIF1α) [37], Heat Shock Transcription Factor 1 (HSF1) [35], Cyclic AMP-Responsive Element-Binding Protein 1 (CREB) [38], Forkhead Box O3 (FOXO3a) [39], Early Growth Response 2 (EGR2) [40], Sterol Regulatory Element Binding Transcription Factor 2 (SREBP2) [41].

AML1 is a leukaemia-associated transcription factor linked with hematopoiesis [42]. It has been identified as an overexpressed gene in the lung of COPD patients [43]. SOX17 is a key transcription factor involved in development that affects the cardiovascular system and several endoderm-derived organs [44]. SOX17 deficiency is linked to pulmonary arterial hypertension (PAH) risk via interactions with Hypoxia Inducible Factor 2 Alpha (HIF-2α) [45]. TAL1 is also a leukaemia associated transcription factor associated with hematopoiesis [45], which can act in complex with LMO2, a LIM-domain protein [46]. ETS-1 is a transcription factor that recognises conserved ETS DNA-binding domains and contributes to cytokine and chemokine regulation. ETS transcription factors are associated with a number of cancers, including breast and leukaemia. ETS-1 is linked to liver fibrosis, where it is thought to be activated by Extracellular Signal-Regulated Kinases (ERK) signalling pathways, matrix remodelling, and cross talk with Transforming Growth Factor (TGF)-Suppressor of Mothers Against Decapentaplegic (SMAD) signalling [47]. Interestingly, there is evidence of significant cross-talk with regard to these transcription factors. For example, ETS-1 and AML-1 can mutually activate DNA binding through the intermolecular interaction of autoinhibitory domains [48], while TAL1 and LMO2 function as complexes, and TAL1, LMO2, and AML-1 (Runx1) are essential for the development of hematopoietic stem cells [49]. It is also notable that these transcription factors are associated with leukaemia and that our THP-1 model is of leukaemic origin. To our knowledge, this is the first evidence suggesting the involvement of any of these five transcription factors in the cellular response to hypercapnia. Of these transcription factors, we focused on ETS-1, based on its association with all nine genes in Figure 4D. We examined ETS-1 and Lamin in nuclear lysates derived from monocytes exposed to buffered hypercapnia for 24 h. Nuclear lamin levels were relatively unchanged in this model, but ETS-1 protein expression in the nucleus was enhanced in hypercapnia. ETS-1 has a predicted molecular weight (MW) of 52 kilodaltons (kD), which is the molecular weight at which we observe our most intense bands using Western blot. We also observe some lower molecular weight bands (with the same pattern of CO_2_-sensitivity), which may be ETS-1 splice variants. p42 and p27 variants of ETS-1 have been described previously [50] and correspond well with the observed molecular weights of the two lower MW bands observed by Western blot (Figure 5A).

In conclusion, our study indicates that *NR4A2* and NR4A3 are CO_2_-responsive transcription factors, as the kinetics of their expression in response to LPS is clearly shifted by exposure to buffered hypercapnia. Furthermore, depletion of *NR4A2* and *NR4A3* led to reduced CO_2_-sensitivity of mitochondrial genes and Hsp-related genes, respectively. However, many other CO_2_-sensitive genes were refractory to depletion of *NR4A2* and *NR4A3*, indicating that the NR4As regulate selective components of the cellular response to buffered hypercapnia but that other transcription factors also contribute. Bioinformatic analysis of conserved CO_2_-sensitive genes implicated several novel putative CO_2_-sensitive transcription factors (AML1, SOX17, TAL1, ETS1, and LMO2), of which ETS-1 was validated to show increased nuclear expression in buffered hypercapnia. Taken together, these data represent a significant resource relating to CO_2_-dependent transcriptional regulation in monocytes and give important insights into the understanding of immune responses in patients experiencing hypercapnia.

## 4. Materials and Methods

### 4.1. Cell Culture

#### 4.1.1. THP-1 Monocytes

THP-1 monocytes were originally sourced from the American Type Culture Collection (ATCC TIB-202) and cultured as described previously [15,26]. Briefly, THP-1 cells were maintained in Roswell Park Memorial Institute (RPMI) 1640 medium supplemented with 10% Foetal Bovine Serum and 1% Penicillin-Streptomycin (ThermoFisher Scientific, Waltham, MA, USA). Cells were maintained at a density of 2 × 10^5^–1 × 10^6^ cells/mL and sub-cultured twice per week. All procedures and treatments prior to cell lysis were performed in a Class II biological safety cabinet or a CO_2_ chamber, as described below. Untransformed THP-1 monocytes were used in Figure 1, Figure 5 and Supplementary Figure S1. All other figures used shNT-THP1, shNR4A2-THP1, and shNR4A3-THP1 monocytes initially described and validated in [25]. Further, validation of knockdown of *NR4A2* and *NR4A3* has been previously described [15] and is also demonstrated in the presence of CO_2_ as part of Supplementary Figure S7.

#### 4.1.2. Hypercapnic Exposures

Hypercapnia exposures were performed as described previously [26]. Briefly, hypercapnic exposures were performed in humidified environmental chambers (Coy Laboratories, Grass Lake, MI, USA) at 37 °C, at 5% or 10% CO_2_ in pH-buffered, pre-equilibrated Dulbecco’s Modified Eagle Medium (DMEM) media [22]. For most THP-1 experiments, cells were used at a density of 7.5 × 10^5^ cells/mL. The maintenance of intracellular and extracellular pH under these conditions has recently been described [26].

#### 4.1.3. RNA-Seq

The RNA-seq protocol was performed as described previously [15,26]. Briefly, total RNA was extracted from cultured cells using the E.Z.N.A. Total RNA Kit I. RNA clean-up was performed (if required) using ethanol precipitation. Subsequent Qubit results determined a RNA integrity number (RIN) score > 9.5 for all samples. Complementary DNA (cDNA) library preparation was performed with polyA selection using Illumina HiSeq, 2 × 150 base pair (bp) configuration, single index, per lane. ~350M raw paired-end reads per lane. Library preparation and sequencing were performed by GeneWiz (Leipzig, Germany). Raw data quality was evaluated with FastQC. Sequence reads were trimmed using Trimmomatic v.0.36 and subsequently mapped to the Homo sapiens GRCh38 reference genome using the STAR aligner v.2.5.2b. Unique gene hit counts were calculated by using featureCounts from the Subread package v.1.5.2. Downstream differential expression analysis was performed using DESeq2 [51]. *p*-values and log2-fold changes were generated using the Wald test. Significant differentially expressed genes (DEGs) were called genes with an adjusted *p*-value (*p*-adj) < 0.05 (Supplementary Tables S1–S4). Initial stringent gene ontology (GO) analysis was performed using GeneSCF v1.1-p2 to generate GO graphs, where a Fisher Exact test was used to determine *p*-values (Supplementary Figure S5). Principal component analysis and read count distribution analysis were performed by GeneWiz (Supplementary Figure S2). Volcano plots of differentially expressed genes were generated using GraphPad Prism, with log2FC on the x-axis and an adjusted *p*-value on the y-axis (Figure 2 and Figure 3). Significant differentially expressed genes (DEGs) were determined as any genes from the DESeq2 workflow that had an adjusted *p*-value (*p*-adj) < 0.05. Nested comparisons for multiple groups were completed by cross-referencing lists of DEGs from pairwise comparisons to generate secondary lists of either common or specific DEGs. Raw data from the RNA-seq experiment is displayed as mean TPM (+/− SEM) to best visualise expression changes between several groups. Statistical comparisons between individual groups used DESeq2 analysis of normalised counts. The bulk RNA-seq data presented in this study are deposited in the Gene Expression Omnibus (GEO) repository, accession number GSE251925.

### 4.2. Quantitative Reverse Transcription Polymerase Chain Reaction (RT-qPCR)

RT-qPCR was performed as described recently [26]. Briefly, RNA was extracted using the EZNA total RNA Kit I (Omega Bio-tek, Norcross, GA, USA) according to the manufacturer’s instructions, and DNase I digestions were performed. cDNA was synthesised using Moloney Murine Leukaemia Virus (M-MLV) Reverse Transcriptase (Promega, Madison, WI, USA). Samples were incubated for 60 min at 37 °C for the cDNA synthesis reaction. cDNA samples were used immediately or stored at −20 °C. cDNA samples were diluted 1 in 4 in nuclease free water. For SYBR qPCR, master mixes for SYBR green reactions ((ThermoFisher Scientific, (Applied Biosystems) Waltham, MA, USA) were prepared using master mix solutions, forward and reverse primers, and nuclease-free water for 10 μL reactions in a 384-well plate. qPCR was performed on an Applied Biosystems RT-PCR machine with the appropriate Applied Biosystems Quant Studio 7 software. For SYBR Green primers, melt curves were generated for each sample for assessment of primer performance. 

Primer details:

*NR4A1* F: gttctctggaggtcatccgcaag R: gcagggaccttgagaaggcca
*NR4A3* F: ccaagccttagcctgcctgtc R: agcctgtcccttactctggtgg
*B-actin* F: cgacaggatgcagaaggaga R:catctgctggaaggtggaca

For TaqMan qPCR, master mixes for TaqMan reactions ((ThermoFisher Scientific, (Applied Biosystems) Waltham, MA, USA) were prepared using master mix solutions, forward and reverse primers, and nuclease free water for 10 μL reactions in a 384-well plate. qPCR was performed on an Applied Biosystems RT-PCR machine with the appropriate Applied Biosystems Quant Studio 7 software.

### 4.3. TaqMan Primer Details

*NR4A2:* Applied Biosystems Hs01117527_g1 (4331182)
*B-actin:* Applied Biosystems (Code:4333762F)

### 4.4. Western Blot

Whole cell protein lysates, Supplementary Figure S1, were prepared using whole cell lysis buffer (150 mM NaCl, 25 mM Tris pH8, 1 mM EDTA, 1% Triton) supplemented with protease inhibitor cocktail (Merck, (Sigma-Aldrich) Darmstadt, Germany, P2714). Lysates were quantified using the DC Protein Assay kit before Sodium Dodecyl-Sulfate Polyacrylamine Gel Electrophoresis (SDS-PAGE) on the Biorad mini-protean system using polyacrylamide gels. Wet transfer was performed onto nitrocellulose membranes and reversibly stained with Ponceau S total protein stain. Immunoblotting was performed with NR4A2 (R&D Systems, Minneapolis MN, USA, PP-N1404-00) or β-actin (Merck, (Sigma-Aldrich) Darmstadt, Germany A5316) primary antibodies. Secondary antibody incubations were performed using Horseradish Peroxidase (HRP)-conjugated anti-mouse antibodies (Cell Signalling Technology, Danvers MA, USA, 7076S). Membranes were incubated with Enhanced Chemi Luminescent Solution (ThermoFisher Scientific, Waltham, MA, USA, 32106) and imaged in a dark room using X-ray film (Fuji Super RX-N, Tokyo, Japan).

Nuclear extracts, Figure 5, were prepared using Buffer A and Buffer C supplemented with protease inhibitor cocktail (Merck, (Sigma-Aldrich) Darmstadt, Germany, P2714) to generate cytoplasmic and nuclear extracts, respectively [29]. Nuclear lysates were quantified using the DC Protein Assay kit before SDS-PAGE on the Biorad (Hercules, CA, USA) mini-protean system using TGX pre-cast gels. Wet transfer was performed onto nitrocellulose membranes and reversibly stained with Revert 700 total protein stain (Li-Cor, Lincoln, NE, USA). Membranes were imaged at 700 nm, washed in Revert 700 wash solution (Li-Cor, Lincoln, NE, USA) and destained in Revert 700 destaining solution (Li-Cor, Lincoln, NE, USA) prior to immunoblotting with the ETS-1 antibody (Cell Signalling Technology, Danvers MA, USA #14069), or Lamin (Cell Signalling Technology, Danvers MA, USA, #4777) antibody. Fluorescent secondary goat anti-mouse or anti-rabbit antibody incubations were performed (DyLight TM 800, ThermoFisher Scientific (Invitrogen), Waltham, MA, USA) and imaged at 800 nm (1:2000 dilution). Membranes were imaged using a LiCor Odyssey Clx imager (Li-Cor, Lincoln, NE, USA) and analysed using EmperiaStudio image analysis software version 2.3.0.154.

### 4.5. Bioinformatic Analysis

Transcription factor interactome analysis was performed on the Metacore platform (MetaCore+MetaDrug^®^ version 20.3 build 70200, Clarivate, London, UK) using the list of DEGs obtained by a fold change cutoff of 1.4 with a padj < 0.05. We have used this approach previously [15,52]. Transcription factors were not directly measured in our data but inferred from gene expression signatures based on an unbiased predictive analysis of known upstream regulators of DEGs. The transcriptional regulators of DEGs were ranked by a Z-score cut-off of 2.0 with a *p*-adj < 0.05. Comparisons were made between shNT 5% CO_2_ vs. shNT 10% CO_2_, shNT 5% CO_2_ +LPS vs. shNT 10% CO_2_ +LPS, shNR4A2 5% CO_2_ +LPS vs. shNR4A2 10% CO_2_ +LPS, shNR4A3 5% CO_2_+LPS vs. shNR4A3 10% CO_2_ +LPS. Supplementary Table S5.

### 4.6. Statistical Analysis

Statistical analysis was performed for the RNA-seq and targeted transcriptomic analysis as described above. ANOVA, or t-tests, were applied as indicated in the figure legends and figures prepared using GraphPad Prism (Boston, MA, USA) (version8).

## Data Availability

Bulk RNA-seq data relating to shNR4A2 and shNR4A3 cells in hypercapnia have been deposited in the GEO repository with accession number GSE251925. Some of the bulk RNA-seq data described in this manuscript has previously been deposited in the Gene Expression Omnibus (GEO) repository. Buffered hypercapnia alters the transcriptional profile in monocytes. Accession number GSE206333 (data relating to shNT cells only) Transcriptional profiling of monocytes deficient in Nuclear Orphan Receptors NR4A2 and NR4A3 reveals distinct signalling roles related to antigen presentation and viral response. Accession number GSE178391 (data relating to shNT, shNR4A2, and shNR4A3 cells in 5% CO_2_ only).

## Supplementary Materials

The following supporting information can be downloaded at: https://www.mdpi.com/article/10.3390/ijms25052852/s1.
