# Supplementary material for: Orphan Nuclear Receptor Family 4A (NR4A) Members NR4A2 and NR4A3 Selectively Modulate Elements of the Monocyte Response to Buffered Hypercapnia
Source: Int J Mol Sci. 2024 Mar 1;25(5):2852. doi: 10.3390/ijms25052852 (PMC10931687; doi:10.3390/ijms25052852)
Supplement: Supplementary file 1 [file ijms-25-02852-s001.zip › 5 ijms-2846400 supplement revision original_EC.pdf]

Supplemental

Supplemental Figure 1

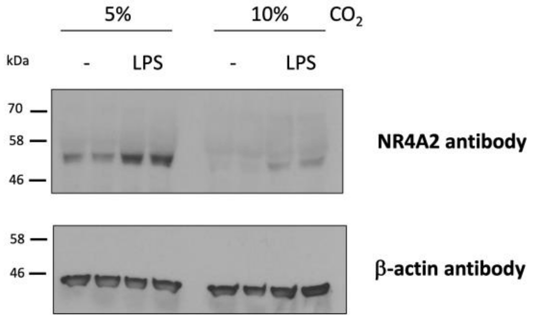

Figure S1. NR4A2 protein is reduced in hypercapnia. Western blot analysis of whole cell lysates from THP-1 monocytes exposed to 5% or 10% CO_2 for 90 mins +/- LPS 2.5µg/ml for an additional 4h. Primary antibodies for NR4A2 and β-actin were used followed by species specific HRP-conjugated secondary antibodies and visualisation using enhanced chemiluminescence. NR4A2 data is representative of *n*=3 separate experiments.

Supplemental Figure 2

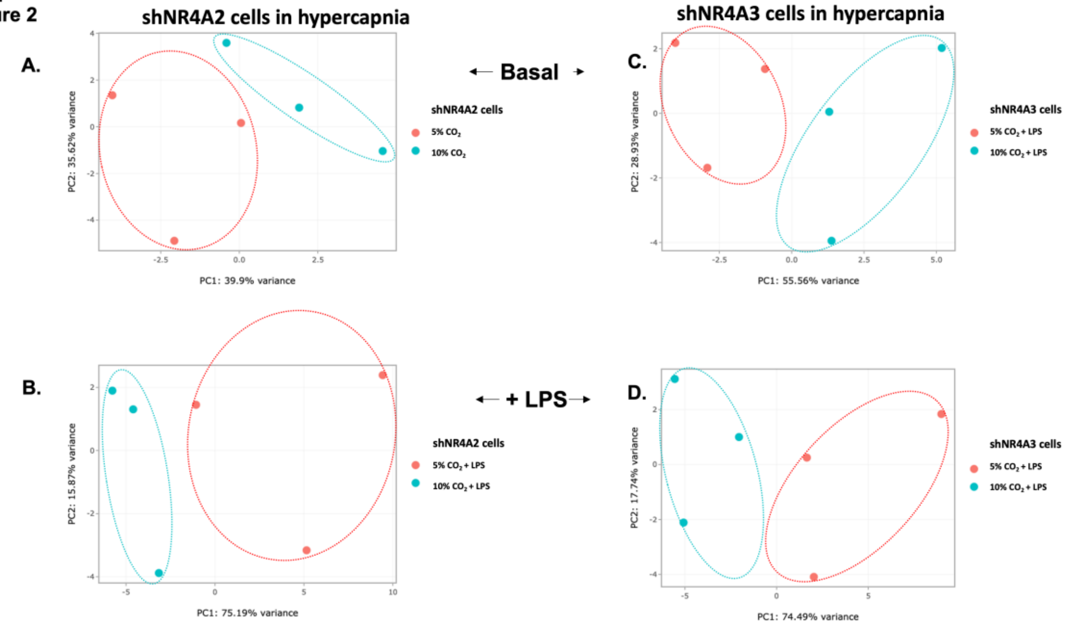

Figure S2. RNA-seq analysis of shNR4A2 and shNR4A3 monocytes exposed to buffered hypercapnia for 4h in the presence of LPS. Principal component analysis of shNR4A2 and shNR4A3 cells exposed to 5% CO_2 (red) or 10% CO_2 (blue) (4h) in the basal (A, C) and LPS stimulated state (2.5µg/ml for 2h) (B, D). The x-axis (PC1) is the vector that displays the most variance between samples and the y-axis (PC2) displays the second most. The percentage of total variance per principal component is shown on the axis label. Data is representative of *n* = 3 individual experiments.

Supplemental
Figure 3

A.

Significant DEGs
shNR4A2 cells
5% CO_2 Vs 10% CO_2

Upregulated genes	LOG2FC	p-adj
AL158166.1	1.513133682	0.01789332
LINC00638	1.289175828	0.01289527
MT-ND4	0.901831302	9.21E-06
MTCO1P12	0.876481578	0.04456635
MANEAL	0.866500856	0.04456635
SERPINB2	0.82893373	0.01789332
MT-ND4L	0.792455979	0.00295093
ZNF74	0.758192403	0.01789332
ALKBH2	0.757463503	0.01789332
MT-CO1	0.750010367	0.00427708
**Downregulated genes**	**LOG2FC**	**p-adj**
**RHBDL3**	-1.23736269	0.02645631
PBX4	-1.230849402	0.04079731
TNF	-1.111081376	0.00186637
RFX8	-1.056672607	0.04079731
ATP2A1	-1.029901667	0.01289527
MIR22HG	-0.917155435	0.0230187
CXCL8	-0.869862187	0.01904809
REC8	-0.868430727	0.0354228
A4GALT	-0.840387807	0.04456635
AL034397.3	-0.820840095	9.21E-06

Basal

B.

Significant DEGs
shNR4A2 cells
5% CO_2 +LPS Vs 10% CO_2 + LPS

Upregulated genes	LOG2FC	p-adj
IGF2	1.886242783	0.00506764
GPR141	1.73612091	0.0046409
ANO5	1.608909746	2.61E-06
FGF2	1.571824613	0.02591089
SLC16A9	1.536446884	8.76E-06
LINC00452	1.534786415	0.00312442
PI15	1.534361143	0.032785
CD80	1.512017558	0.00411919
NR5A2	1.500300244	0.04000766
AL133330.1	1.500081302	0.01510462
**Downregulated genes**	**LOG2FC**	**p-adj**
KCNK13	-1.816902654	0.01039153
B3GNT7	-1.662201722	1.94E-05
TMEM37	-1.514588968	0.00033047
PDK4	-1.462895076	0.02544185
SMIM3	-1.448126654	3.66E-05
**RHBDL3**	-1.419928648	0.00428612
WNT7B	-1.407503797	3.56E-05
F2RL3	-1.26111394	0.0021081
ST7-AS1	-1.243265224	0.04401988
GPRC5C	-1.242618146	0.00013872

+ LPS

Figure S3. Top 10 differentially regulated genes in response to buffered hypercapnia in shNR4A2 cells. List of the 10 most differentially expressed genes cells (up (green) and down (red)) in shNR4A2 in hypercapnia in the basal (A) and LPS stimulated state (2.5µg/ml for 2h) (B). Genes are ranked by Log2 fold change. All genes have a *p*-adj<0.05. Data is representative of *n* = 3 individual experiments.

Supplemental
Figure 4

A.

Significant DEGs
shNR4A3 cells
5% CO_2 Vs 10% CO_2

Upregulated genes	LOG2FC	p-adj
RN7SL2	1.93945221	0.011400512
SLC16A9	1.51550278	0.000224193
THNSL1	1.15787347	0.024101935
LINC00638	1.09600166	0.005308471
SLC46A1	0.99804242	0.014229174
HSPH1	0.9498798	3.26257E-07
RIOX2	0.87844398	4.30557E-06
SERPINB2	0.84603628	6.61351E-06
TBC1D9	0.83792635	0.003106565
HSPA4L	0.80506854	0.000224193
**Downregulated genes**	**LOG2FC**	**p-adj**
**C2orf48**	-1.2339701	0.003001251
ATP2A1	-1.2063019	0.000154311
MIR22HG	-1.1571433	0.000734399
AP001347.1	-1.1266397	0.040724478
AC114490.1	-1.1070416	0.041340928
RFX8	-1.092369	0.00102008
ARTN	-1.0088844	0.00060915
MAFF	-1.0008972	0.010459013
OSM	-0.952871	0.00533492
RAB43	-0.9289324	0.017810107

Basal

B.

Significant DEGs
shNR4A3 cells
5% CO_2 +LPS Vs 10% CO_2 + LPS

Upregulated genes	LOG2FC	p-adj
SLAMF1	1.8364275	0.00215068
SPATA31C1	1.77899834	0.00014948
CRIM1	1.767316	0.03020285
IGKC	1.76586563	0.00023239
C6orf58	1.64356511	0.00070974
EVA1A	1.59751902	6.47E-05
HSPA1A	1.5590445	0.01313429
RPA4	1.48803857	0.0010438
PSTPIP2	1.43967736	0.00963072
TP63	1.43036218	0.00322725
**Downregulated genes**	**LOG2FC**	**p-adj**
AC116351.2	-1.7062145	0.00023526
B3GNT7	-1.5215211	0.03358237
F2RL3	-1.4876489	0.00011359
AC132872.3	-1.4788048	0.00160728
AC132872.4	-1.4588767	6.47E-05
**C2orf48**	-1.4494994	0.00084597
OCLN	-1.3251797	0.04307551
FAM241B	-1.2868822	0.0097268
ADAMTS15	-1.277873	0.00363948
SLC6A6	-1.2532132	2.52E-10

+ LPS

Figure S4. Top 10 differentially regulated genes in response to buffered hypercapnia in shNR4A3 cells. List of the 10 most differentially expressed genes cells (up (green) and down (red)) in shNR4A3 in hypercapnia in the basal (A) and LPS stimulated state (2.5µg/ml for 2h) (B). Genes are ranked by Log2 fold change. All genes have a *p*-adj<0.05. Data is representative of *n* = 3 individual experiments.

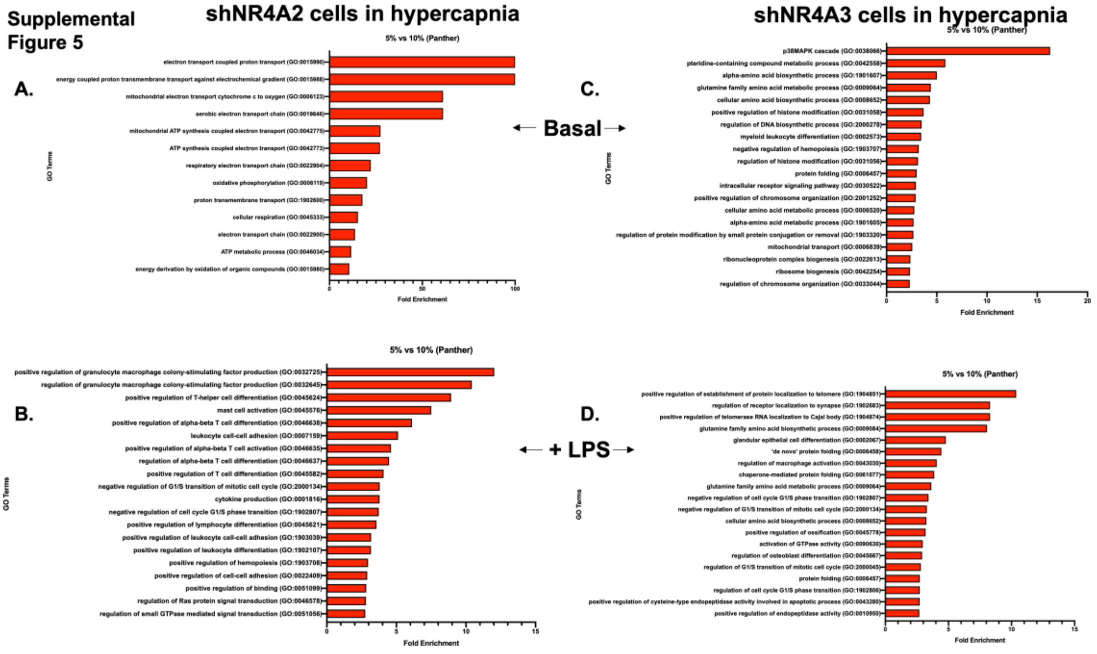

Figure S5. GO analysis of shNR4A2 and shNR4A3 cells exposed to hypercapnia in the basal and LPS-stimulated state. List of the top gene ontology terms associated with significantly differentially expressed genes in hypercapnia in the basal (A, C) and LPS stimulated state (2.5µg/ml for 2h) (B, D) in shNR4A2 and shNR4A3 cells, using a cut off value for significance of *p*-adj<0.05. Terms are ranked by fold enrichment. Data is representative of *n* = 3 individual experiments.

Supplemental Figure 6

A.

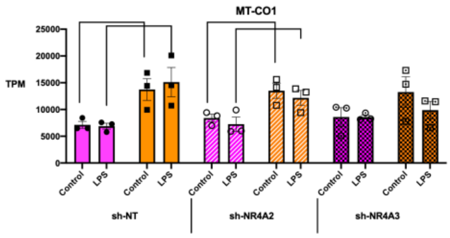

B.

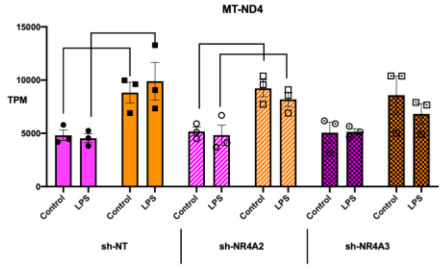

C.

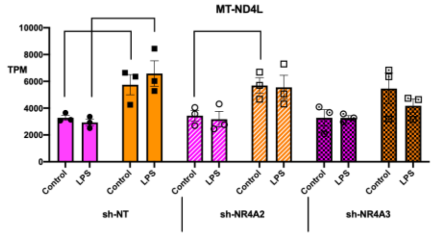

Figure S6. RNA-seq analysis of mitochondrial genes in shNT, shNR4A2 and shNR4A3 cells exposed to hypercapnia in the basal and LPS-stimulated state. Raw TPM values extracted from RNA-seq data for (A) *MT-CO1*, (B) *MT-ND4*, and (C) *MT-ND4L* in shNT, shNR4A2 and shNR4A3 THP-1 cells in the basal (unstimulated) and LPS stimulated (2.5µg/ml for 2h) state in both 5% (pink) and 10% (orange) CO_2 conditions (4h). CO_2 comparisons that had a *p*-adj<0.05 are indicated by the brackets. Data shown as mean +/- SEM for *n*=3 independent experiments.

Supplemental Figure 7

A.

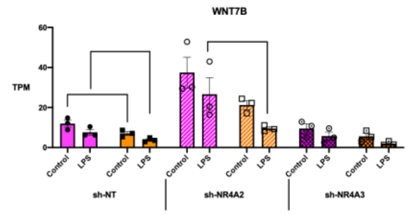

B.

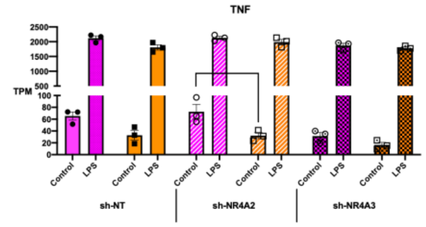

C.

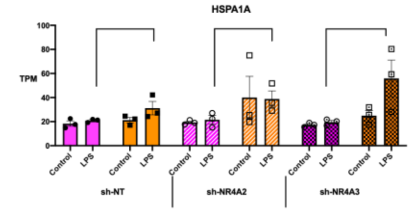

D.

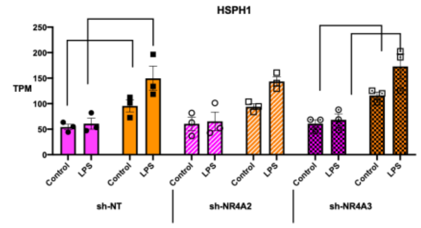

Figure S7. RNA-seq analysis of inflammation and heat-shock associated genes in shNT, shNR4A2 and shNR4A3 cells exposed to hypercapnia in the basal and LPS-stimulated state. Raw TPM values extracted from RNA-seq data for (A) *WNT7B*, (B) *TNF*, (C) *HSPA1A* and (D) *HSPH1* in shNT, shNR4A2 and shNR4A3 THP-1 cells in the basal (unstimulated) and LPS stimulated (2.5µg/ml for 2h) state in both 5% (pink) and 10% (orange) CO_2 conditions (4h). CO_2 comparisons that had a p-adj<0.05 are indicated by the brackets. Data shown as mean +/- SEM for *n*=3 independent experiments.

Supplemental Figure 8

	GO Term	shNT	shNR4A2	shNR4A3	
**Basal**	electron transport coupled proton transport (GO:0015990)	√	√		Lost in shNR4A3 cells
	ATP synthesis coupled electron transport (GO:0042773)	√	√		Lost in shNR4A3 cells
	cellular respiration (GO:0045333)	√	√		Lost in shNR4A3 cells
	alpha-amino acid biosynthetic process (GO:1901607)	√		√	Lost in shNR4A2 cells
	protein folding (GO:0006457)"	√		√	Lost in shNR4A2 cells
	ribosome biogenesis (GO:0042254)	√		√	Lost in shNR4A2 cells
	ribonucleoprotein complex biogenesis (GO:0022613)	√		√	Lost in shNR4A2 cells
**+ LPS**	positive regulation of establishment of protein localization to telomere (GO:1904851)	√		√	Lost in shNR4A2 cell
	positive regulation of telomerase RNA localization to Cajal body (GO:1904874)	√		√	Lost in shNR4A2 cells
	'de novo' protein folding (GO:0006458)	√		√	Lost in shNR4A2 cells
	negative regulation of G1/S transition of mitotic cell cycle (GO:2000134)		√	√	Gained in shNR4A2/A3 cells
	negative regulation of cell cycle G1/S phase transition (GO:1902807)		√	√	Gained in shNR4A2/A3 cells

Figure S8. Comparison of common CO_2-responsive GO terms between shNT, shNR4A2 and shNR4A3 cells exposed to hypercapnia in the basal and LPS-stimulated state. Analysis reveals enriched GO terms that are lost in shNR4A3 cells (green text), lost in shNR4A2 cells (orange text) and gained in shNR4A2/3 cells (black text).

Supplemental Figure 9

A.

B.

C.

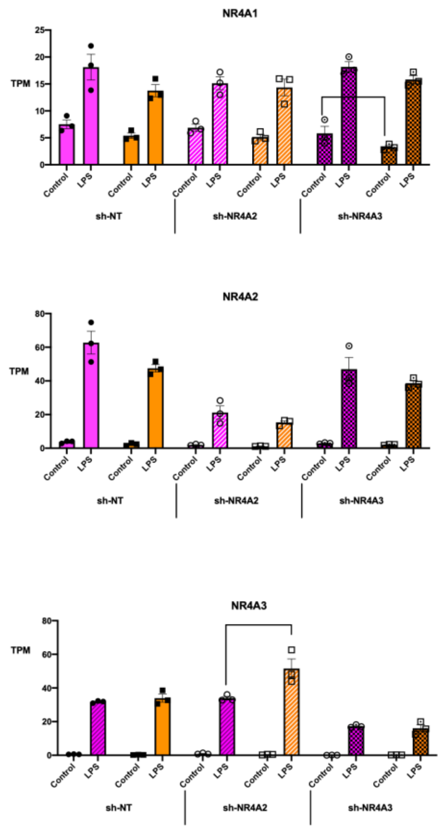

Figure S9. RNA-seq analysis of orphan nuclear receptor family 4A genes in shNT, shNR4A2 and shNR4A3 cells exposed to hypercapnia in the basal and LPS-stimulated state. Raw TPM values extracted from RNA-seq data for (A) *NR4A1*, (B) *NR4A2*, and (C) *NR4A3* in shNT, shNR4A2 and shNR4A3 THP-1 cells in the basal (unstimulated) and LPS stimulated (2.5µg/ml for 2h) state in both 5% (pink) and 10% (orange) CO_2 conditions (4h). CO_2 comparisons that

had a p-adj<0.05 are indicated by the brackets. Data shown as mean +/- SEM for n=3 independent experiments.

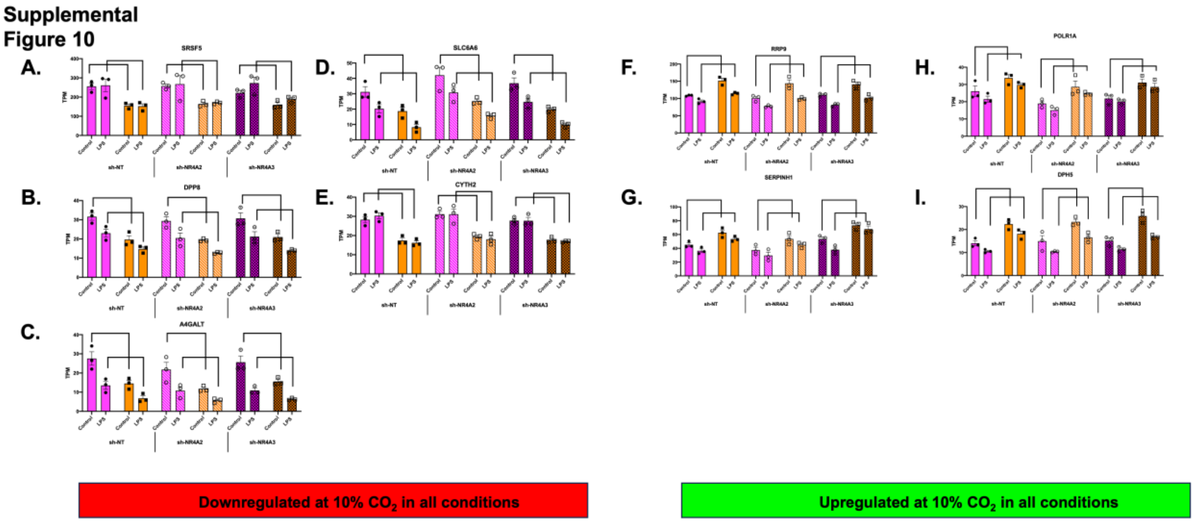

Figure S10. Hypercapnia consistently regulates genes in shNT, shNR4A2 and shNR4A3 THP-1 monocytes in the presence and absence of LPS. Raw TPM values extracted from RNA-seq data for (A) *SRSF5*, (B) *DPP8*, (C) *A4GALT*, (D) *SLC6A6*, (E) *CYTH2* (F) *RRP9*, (G) *SERPINH1*, (H) *POLR1A* and (I) *DPH5* in shNT/shNR4A2/shNR4A3 THP-1 cells in the basal and LPS stimulated (2.5µg/ml for 2h) state in both 5% and 10% CO_2 conditions (4h). All CO_2 comparisons had a p-adj<0.05 as indicated by the brackets. Data shown as mean +/- SEM for n=3 independent experiments.
